# Bone and lymph node metastases from occult mammary carcinoma: a case report of carcinoma of unknown primary (CUP) Syndrome

**DOI:** 10.1259/bjrcr.20190064

**Published:** 2019-11-15

**Authors:** Antonino Cattafi, Mariacarmela Santarpia, Martina Francesca Micalizzi, Carmelo Sofia, Elvira Condorelli, Alessia Dottore, Giuseppe Altavilla, Alfredo Blandino, Giorgio Ascenti, Maria Adele Marino

**Affiliations:** 1 Department of Biomedical Sciences and Morphologic and Functional Imaging, Policlinico Universitario G. Martino, University of Messina, Messina, Italy; 2 Department of Human Pathology of Adult and Evolutive Age "G. Barresi" Medical Oncology Unit, University of Messina, Messina, Italy

## Abstract

Cancer of unknown provenance is a rare disease, accounting approximately for up to 1% of all breast cancers. A 68-year-old female was admitted to the Medical Oncology Unit of Policlinico Universitario G.Martino

because of diffused bone-involvement, with mixed (osteolytic/osteoblastic) features, which interested almost every skeletal structure of the body (vertebral bodies of the entire column, costal skeleton, sternum, proximal third of both humeri, scapulae, clavicles, pelvis and femurs), suspicious for metastatic disease.

Cancer of unknown primary (CUP) is rare, accounting for up to 1% of all breast cancers.^1^ We refer to the term “CUP syndrome” when cancer cells have spread and metastasized in the body but the primary site of cancer is unknown and/or cannot be identified.^2^ Breast cancer may be especially suspected when axillary nodes are involved, but mammography and ultrasound are often negative. Breast MRI is recommended by international guidelines in case of findings suspicious for CUP syndrome^1,3^ which enables the detection of an occult primary breast cancer in 35–100% of cases. If breast MRI is negative, immediate surgery may be avoided.^1,3^

## Case report

A 68-year-old female was admitted at the Medical Oncology Unit of our Hospital because of diffused bone-involvement, with mixed (osteolytic/osteoblastic) features suspicious for metastatic disease, occasionally discovered during an abdominal CT scan performed for stipsis at the Emergency Department of another Hospital.

**Figure 1.**
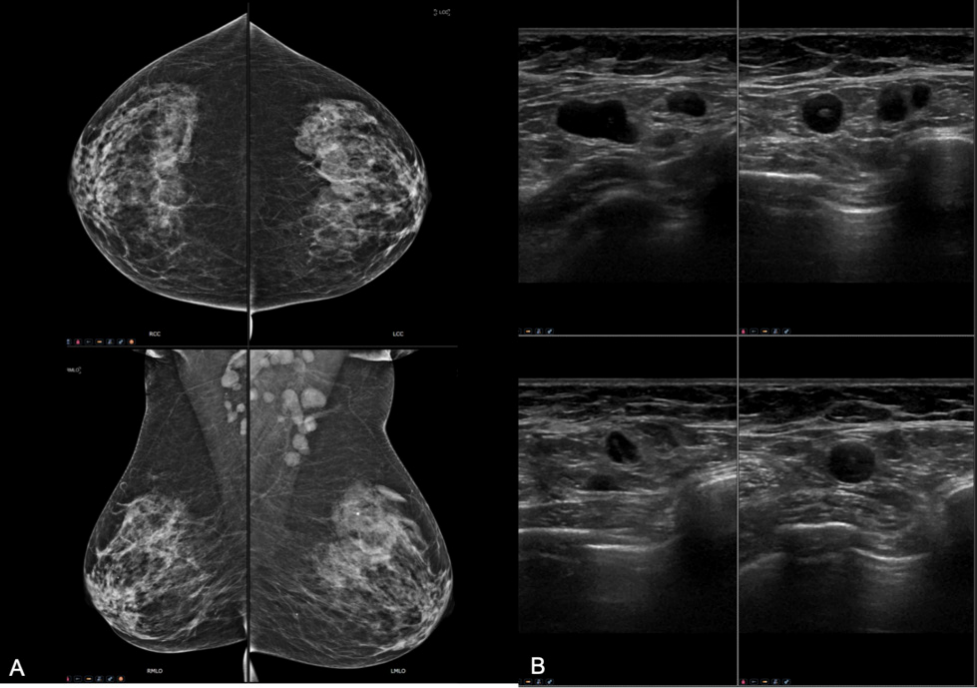
Breast work-up including four complete view mammography (A) and ultrasound (B). No mass nor suspicious microcalcifications. Both examinations showed enlarged bilateral suspicious lymph-nodes in both axilla. The need of an histologic samples of the lymphnode is needed (BI-RADS 4c).

The patient demonstrated an increase in blood markers, namely CA 15.3 (125 UI/ml), CA 125 (272 UI/ml), CEA (32 ng ml^−1^), but at colonoscopy, mammography and breast ultrasound no significant findings were found with the exception of multiple enlarged and suspicious bilateral axillary lymph nodes (Figure 1).

**Figure 2.**
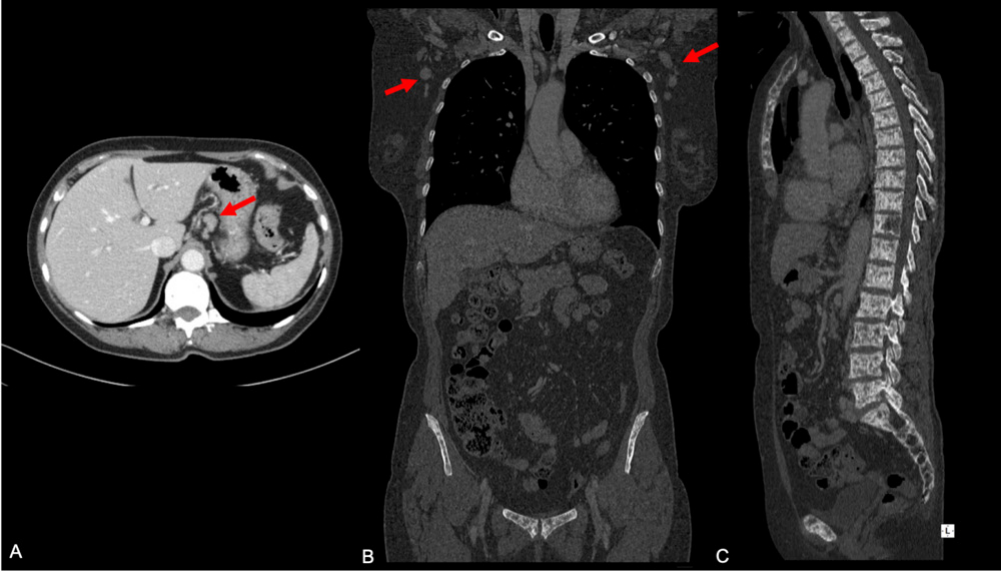
Contrast-enhanced body CT. (A) axial, (B) coronal and (C) sagittal plane showing suspicious node in the abdomen and in the axilla (A, B). It also shows (B, C) diffuse bone-involvement, with mixed both osteolytic and osteoblastic features, which interested almost every skeletal structures of the body (vertebral bodies of the entire column, costal skeleton, sternum, proximal third of both humeri, scapulae, clavicles, pelvis and femurs).

**Figure 3.**
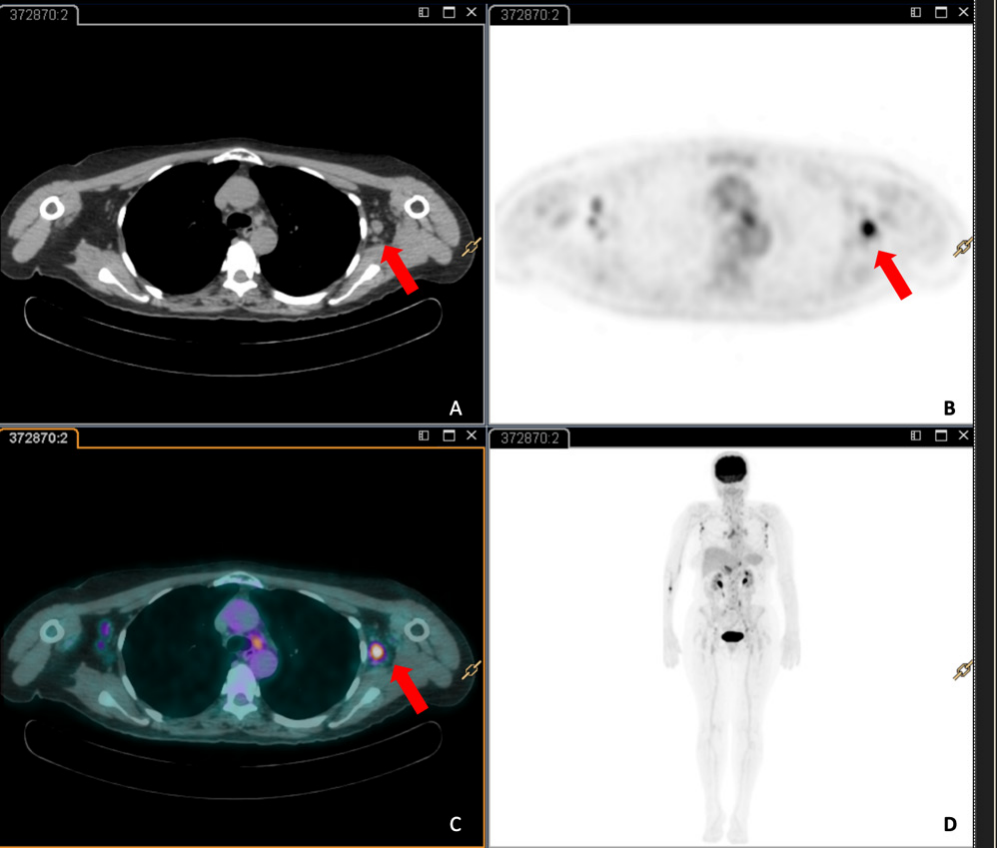
Axial view of total body CT (A), PET imaging (B), the hybrid PET/CT (c) and coronal view of total body PET (D). The images (A) shows an enlarged node in the left axilla (red aarow) which demonstrates abnormal metabolic activity (B, C) (red arrows). PET, positron emission tomography.

Contrast-enhanced body-CT scan was undertaken and revealed multiple suspicious lymph nodes in several mediastinal and abdominal lymphatic stations (Figure 2). Furthermore, it confirmed numerous enlarged nodes in both axilla, with a prominent suspicious node in the left side (20 mm). To better define these CT findings, a PET/CT scan was performed. PET/CT showed high metabolic activity of the lymphadenopathies especially in the left axilla (SUV ranged between 7.41 and 9.10) with a significant uptake of the enlarged 20 mm node of the left axilla (Figure 3).

**Figure 4.**
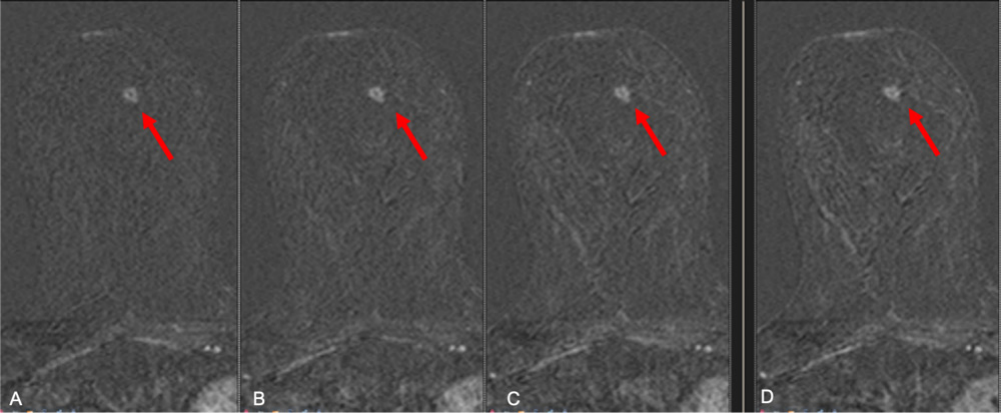
MRI of the right breast. *T*_1_weighted fat sat subtracted at 2 min (A), 4 min (B), 6 min (C) and 8 min (D) after administration of contrast media. There is a 9 mm non-mass enhancing lesion, with in-homogenous uptake of contrast media which is persistent overall the dynamic phase.

**Figure 5.**
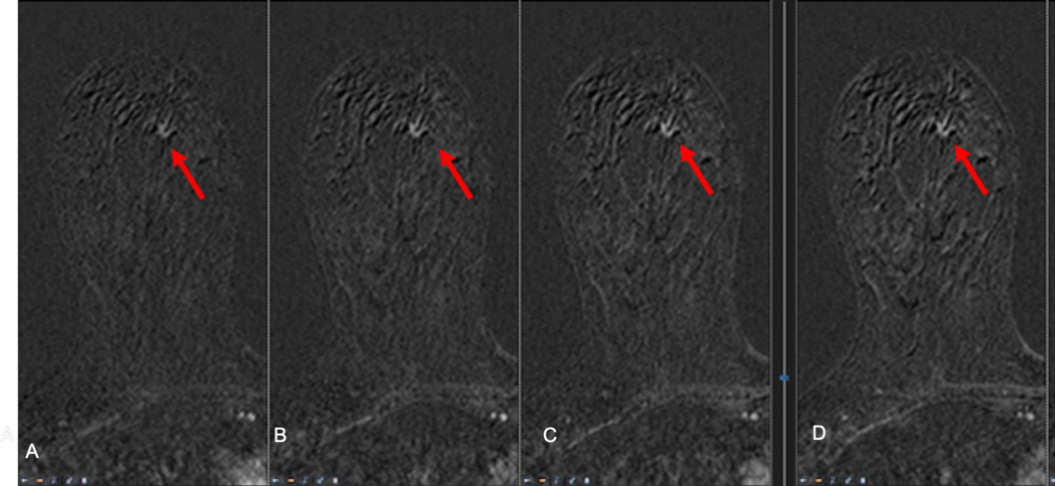
MRI of the right breast after 4 months from the prior examination (Figure 4) and four cycles of docetaxel + trastuzumab and pertuzumab. *T*_1_ weighted fat sat subtracted at 2 min (A), 4 min (B), 6 min (C) and 8 min (D) after administration of contrast media. The small 9 mm non-mass enhancing lesion is now less visible showing a different uptake of contrast media which is now slow and progressive overall the dynamic examination. The is stable in size (5–6 mm).

Therefore, a biopsy of the left axillary node was performed with the final result of cancer cells of breast origin (ER 95%, PGR 5%, Her 3+, Mib-1 5%). Therefore, following the International recommendations,^1,3^ we performed a breast MRI that showed a focal non-mass lesion of about 9 mm in the inner-medial region of the right breast (BI-RADS 4) and the bilateral axillary lymphadenopathies (Figure 4). No correlation was found at a second-look ultrasound. The patient started first-line treatment with docetaxel + trastuzumab and pertuzumab. A new breast MRI was performed at the end of the third cycle of treatment (Figure 5). The lesion was stable lesion in size with a decrease in enhancement. Therefore, the patient underwent to a breast MRI biopsy in another facility with the pathological diagnosis of usual ductal hyperplasia which was confirmed at the final histological evaluation.

## Discussion

Up to 4% of newly diagnosed cases of malignant neoplasms involve CUP, representing the fourth most common cause of cancer death in both males and females. Usually, the first manifestations of CUP consist in metastases to lymph nodes, lungs, liver or bones and the primary tumor can be detected in up to 40% of patients. CUP syndrome still have a poor prognosis, with a median of 2 year survival in patients with disseminated disease.^4^ CUP syndrome includes a multitude of possible differential diagnosis, such as lymphoma, melanoma and lung cancer.^5^

CUP should be with a comprehensive evaluation, involving several specialized figures, that can manage all its diagnostic and therapeutic phases.

## Learning points

- Usually, the first manifestations of CUP consist in metastases to lymph nodes, lungs, liver or bones and the primary tumor can be detected in up to 40% of patients.
- Breast MRI is recommended by international guidelines in case of findings suspicious for CUP syndrome.
- CUP should be with a comprehensive evaluation, involving several specialized figures.
